# Impaired Thermogenesis and a Molecular Signature for Brown Adipose Tissue in* Id2* Null Mice

**DOI:** 10.1155/2016/6785948

**Published:** 2016-04-10

**Authors:** Peng Zhou, Maricela Robles-Murguia, Deepa Mathew, Giles E. Duffield

**Affiliations:** Department of Biological Sciences and Eck Institute for Global Health, University of Notre Dame, Notre Dame, IN 46556, USA

## Abstract

Inhibitor of DNA binding 2 (ID2) is a helix-loop-helix transcriptional repressor rhythmically expressed in many adult tissues. Our previous studies have demonstrated that* Id2* null mice have sex-specific elevated glucose uptake in brown adipose tissue (BAT). Here we further explored the role of* Id2* in the regulation of core body temperature over the circadian cycle and the impact of* Id2 *deficiency on genes involved in insulin signaling and adipogenesis in BAT. We discovered a reduced core body temperature in* Id2*−/− mice. Moreover, in* Id2*−/− BAT, 30 genes including* Irs1*,* PPAR*s, and* PGC-1*s were identified as differentially expressed in a sex-specific pattern. These data provide valuable insights into the impact of* Id2 *deficiency on energy homeostasis of mice in a sex-specific manner.

## 1. Introduction

The circadian clock is an autoregulatory network that regulates behavioral and metabolic programing in the context of a 24 h light-dark (LD) cycle [1]. Body temperature is one of the representing benchmarks of circadian patterning, which peaks in animals while awake and troughs while asleep [2]. Brown adipose tissue (BAT) is a major site for rodent thermogenesis, due to its involvement in controlling circadian thermogenic rhythms and influencing adaptability to environmental temperature challenges [3]. A previous study has revealed rhythmic expression patterns of over 5,000 genes in murine BAT, including genes associated with the circadian clock, adipose function, and metabolism [4]. Moreover, glucose uptake in BAT exhibits a diurnal rhythm [5].

The* Inhibitor of DNA binding 2* (*Id2*) gene encodes a helix-loop-helix (HLH) transcriptional regulator, which is rhythmically expressed in many mammalian tissues and involved in the input pathway, core clock function, and output pathways of the circadian clock [6–9]. Our previous studies have shown that* Id2−/−* mice exhibit lower levels of locomotor activity, extended nighttime activity patterns of feeding and locomotor activity, and sex- and age-dependent enhanced glucose tolerance and insulin sensitivity [10]. Moreover, an energy-rich diet is able to rescue the disturbances to metabolic homeostasis and survival in the* Id2*−/− mice sex-specifically [11]. Importantly,* Id2−/−* mice show a sex-dependent elevated glucose uptake in interscapular BAT (iBAT) [10].* Id2* also plays a role in white adipose tissue (WAT) adipogenesis [10–12]. However, the role of* Id2* on temperature homeostasis regulation and its influence on BAT physiology remain unknown. Therefore, we investigated the function of* Id2* in the regulation of temperature rhythms under normal and thermoneutral conditions in a sex-specific manner and also profiled the expression of genes involved in insulin signaling and adipogenesis in BAT of* Id2−/−* mice, sex-specifically.

## 2. Materials and Methods

### 2.1. Animals

The generation of* Id2*−/− mice and genotype determination were performed as described previously [7, 10, 11]. Mice were maintained on a regular chow diet and sterile water containing antibiotic* ad libitum* [7, 10, 11]. All mice were housed in laboratory cages at normal temperature (21°C ± 1°C) and humidity of 50–65% under a 12 : 12 light : dark (LD) cycle with lights on at Zeitgeber time (ZT) 0 and lights off at ZT12. Controls were age- and sex-matched WT littermate mice. Animal experiments were approved by the University of Notre Dame Animal Care and Use Committee (Protocol number 14-02-1559) and performed in accordance with NIH Guidelines for the Care and Use of Laboratory Animals.

### 2.2. Temperature Measurement

Temperature measurements were carried out on 2-month–1.5-year- (5.5-month median) old male and female* Id2−/−* mice and WT littermates, housed individually in a climate-controlled room set to either normal (21°C ± 1°C) or thermoneutral (30°C ± 1°C) temperature. Body temperature sampling was conducted at 3 h intervals over the 24 h LD cycle. For thermoneutral conditions measurement, all WT and* Id2−/−* mice used in the studies were allowed to acclimate to thermoneutral temperature for 1 week before temperature measurement. Core body temperature was measured using subcutaneously surgically implanted telemetric transmitters positioned proximal to the iBAT (IPTT 300 transponders, Bio Medic Data Systems, Seaford, DE) following isoflurane anesthetization [3]. After a week of recuperation, core temperatures were recorded over a 24 h period.

### 2.3. iBAT PCR Array Preparation and Analysis

iBAT tissue was harvested at ZT16 (*Id2* mRNA circadian rhythm in iBAT has a broad peak phase between ZT8 and ZT16) [4].* Id2−/−* and WT male (WT = 8,* Id2−/−* = 6) and female (WT = 6,* Id2−/−* = 4) mice from 3–9 months (6.1-month median) were sacrificed and iBAT tissue was frozen in liquid nitrogen and stored at −80°C until analyzed. RNA extraction was performed as described previously [7, 13]. We also measured iBAT weight of these and additional mice (3–10-month-old* Id2−/−* mice and WT littermates; 6.3-month median; male, WT = 15,* Id2−/−* = 7; female, WT = 10,* Id2−/−* = 9) as described previously [10, 11]. Total RNA was purified following a Trizol extraction and sodium acetate/ethanol treatment. RNA integrity was assessed using a Bioanalyzer 2100 (Agilent Technologies, Santa Clara, CA). RNA was subjected to a DNASe I treatment, and cDNA was synthesized by RT^2^ First Strand Kit (SABiosciences). Relative mRNA expression of 168 genes involved in insulin signaling and adipogenesis pathways was determined by using the mouse PCR arrays (PAMM-030ZC-24 and PAMM-049ZC-24, SABiosciences). Quantitative real-time PCR was performed using an Applied Biosystems 7500 system with RT2 SYBR green ROX qPCR master mix reagent (Qiagen). PCR array data were calculated by the comparative cycle threshold method and analyzed by Web-based free PCR array data analysis software provided by SABiosciences. Normalization of expression was to housekeeping genes provided on each array (*Actb*,* B2m*,* Gapdh*,* Gusb*, and* Hsp90ab1*). Clock controlled genes (CCGs) were identified from the CIRCA database of Mouse 1.OST Brown Adipose (Affymetrix) (http://bioinf.itmat.upenn.edu/circa/) where we defined CCGs as a JTK_CYCLE algorithm determined *q* < 0.1 value and a period length of 20–28 h as described previously [13–15]. Circadian phase was determined from the Lomb-Scargle phase values within CIRCA.

### 2.4. Statistics

Data were analyzed using Sigma Plot 12.0 software to run two-factor ANOVA. Where necessary, data were ranks transformed to correct for nonnormal distributions. The linear regression of iBAT temperature-body weight relationship was generated and analyzed using Prism 5.0 Graphpad software. PCR array data were analyzed using the Web-based free PCR array data analysis software provided by SABiosciences (Student's *t*-test).

## 3. Results

### 3.1. Loss of* Id2* Results in a Reduced Core Body Temperature in Male and Female Mice

**Figure 1:**
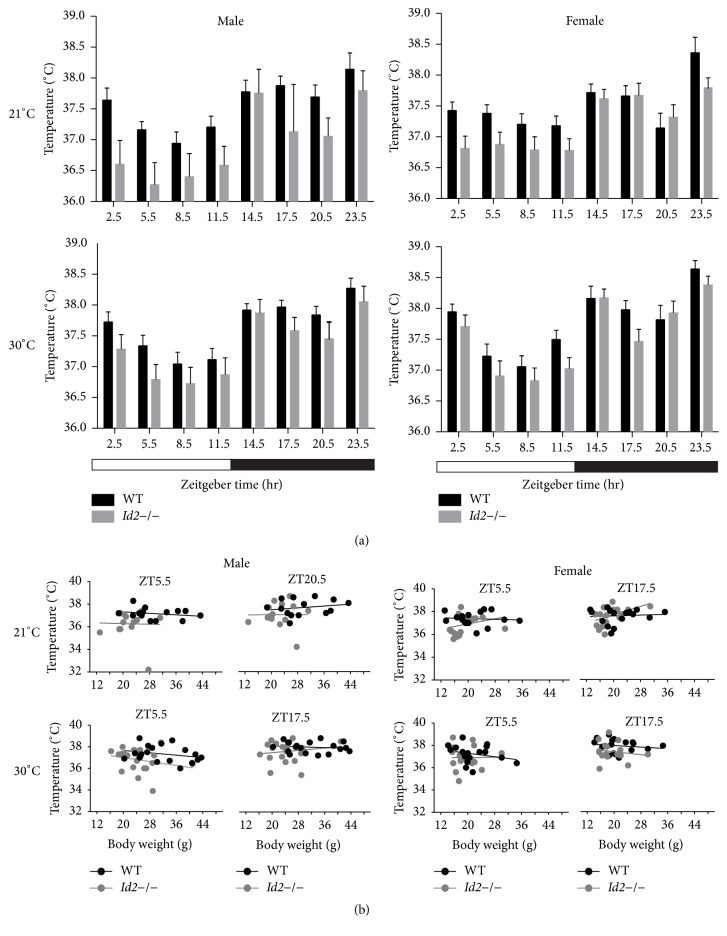
Sex-specific regulation of body temperature in* Id2*−/− mice. (a) Body temperature measurements of WT and* Id2−/−* male (left) and female (right) mice under normal temperature (upper panel) or thermoneutral temperature (lower panel) over 24 hrs. Values are mean ± SEM. Two-factor ANOVA was performed. ANOVAs revealed significantly lower body temperatures for both male and female* Id2*−/− mice compared to WT mice. The genotypic effect was independent of the prevailing ambient temperature. (b) Upper: regression analysis of body weight to body temperature of WT and* Id2−/− *mice under normal ambient temperature (left: male at ZT5.5 and ZT20.5; right: female at ZT5.5 and ZT17.5). Lower: regression analysis of body weight to body temperature of WT and* Id2−/− *mice under thermoneutral temperature conditions (left: male at ZT5.5 and ZT17.5; right: female at ZT5.5 and ZT17.5). Values are individual animal body temperatures and their respective measures of body mass. Note that no linear regression was found to be significant (n.s.), indicating that body mass does not predict body temperature for any group analyzed, examined under either 21°C or 30°C environmental temperatures.

**Figure 2:**
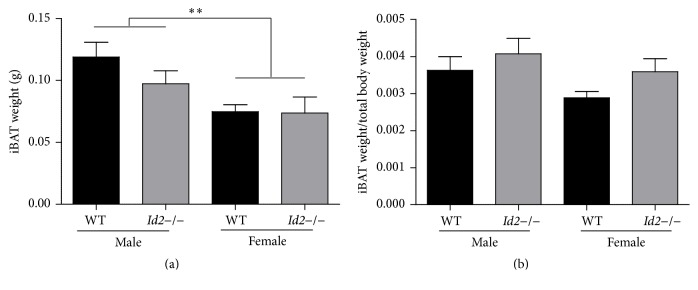
Brown Adipose tissue weight in* Id2*−/− mice. (a) Interscapular brown adipose tissue (iBAT) mass (g) from WT and* Id2−/−* mice. (b) Ratio of weight of iBAT tissue to total body mass from WT and* Id2−/−* mice. Values are mean ± SEM. Two-factor ANOVAs were performed followed by Tukey's post hoc tests, ^*∗∗*^ *p* < 0.01. No significant differences were observed between groups in the iBAT mass/body mass analysis.

The discovery of a diurnal rhythm of glucose uptake in mice iBAT and a sex-dependent elevated glucose uptake in iBAT of* Id2−/−* mice prompted us to investigate whether* Id2* contributes to thermoregulation [5, 10]. At normal ambient temperature conditions (21°C), ablation of* Id2* reduced core body temperature across the 24 h day, in both male and female mice (Figure 1(a)) (males, wild types (WTs) = 14,* Id2−/−* = 14, ANOVA, time (T), *p* < 0.001, genotype (G) *p* < 0.001, interaction (I), n.s.; females, WTs = 18,* Id2−/−* = 17, ANOVA, T, *p* < 0.001, G, *p* < 0.05, I, n.s.). Considering the possibility of any confounding genetic background contribution and partial stimulation of BAT activity occurring under normal temperature conditions,* Id2−/−* mice core body temperature was also measured under thermoneutral conditions (30°C) [3, 16]. Consistently, at thermoneutrality,* Id2−/−* mice displayed a reduced core body temperature (Figure 1(a)) (males, WTs = 19,* Id2−/−* = 20, ANOVA, T, *p* < 0.001, G, *p* < 0.01, I, n.s.; females, WTs = 18,* Id2−/−* = 17, ANOVA, T, *p* < 0.001, G, *p* < 0.01, I, n.s.). Under both conditions and in both sexes, no interaction between time and genotype was discovered, suggesting a generalized effect of the null mutation on core body temperature rather than a time-of-day specific contribution of the gene deletion. Regression analysis of time-of-day representative core body temperatures (day or night) revealed no significant relationships between temperature and body mass for either* Id2*−/− or WT mice. However,* Id2*−/− mice of both sexes showed consistently lower *y*-intercept lines compared to WT mice when examined during either the daytime (ZT5.5) or nighttime (ZT17.5 or ZT20.5), thus confirming the consistently lower temperature of the* Id2* null mice (Figure 1(b); Supplementary Table  1 in Supplementary Material available online at http://dx.doi.org/10.1155/2016/6785948). Lastly,* Id2−/−* mice exhibited no statistically significant difference in iBAT weight and iBAT to body weight ratio compared to WT controls (Figures 2(a) and 2(b)) (two-factor ANOVAs, iBAT weight, G, n.s., sex (S), *p* < 0.01, I, n.s.; iBAT/body weight ratio, G, n.s., S, n.s., I, n.s.). The mean and SEM body mass of WT and* Id2*−/− mice for both iBAT weight and body temperature experiments are shown in Supplementary Table  2: note that both male and female* Id2*−/− mice had on average a lower body mass compared to WT counterparts (two-factor ANOVA, G, *p* < 0.001, S, *p* < 0.001, I, n.s.).

### 3.2. Sex-Specific Differential Gene Expression Associated with Insulin Signaling and Adipogenesis in iBAT of* Id2−/−* Mice

Our previous results showed sex-dependent enhanced insulin sensitivity and glucose uptake in iBAT of* Id2−/−* mice [10]. In the current study we observed a decreased core body temperature in* Id2−/−* mice as described above. To fully evaluate the impact of ablation of* Id2* on BAT gene-regulation, we performed a gene expression analysis using RT^2^ Profiler PCR Arrays of BAT derived from* Id2−/−* mice and their WT littermates collected at the same time of the 24 h day (specifically ZT16). Deferentially regulated genes involved in insulin signaling and adipogenesis are shown in Tables 1 and 2, respectively. Thirty of 168 genes examined were identified as differentially expressed when analyzed as a cohort or as individual sex-specific groups. Using the CIRCA database as a resource [14], six genes were identified as clock controlled genes (CCGs), of which four oscillate in proximal phase with the rhythm of* Peroxisome proliferator activated receptor alpha* (*Pparα*), peaking during the middle of the day (~circadian time (CT) 6; CT12 = onset of night in prior LD cycle). Of importance for insulin signaling,* glucose-6-phosphatase*,* catalytic* (*G6pc*), was upregulated in* Id2−/−* females and the related G6pc family member* G6pc2* downregulated in* Id2−/−* males (*p* = 0.079, approaching significance) compared to WTs.* Insulin receptor substrate 1* (*Irs1*) was upregulated in both male and female* Id2−/−* mice.* Protein Kinase C*,* iota* (*Prkcι*), was downregulated in* Id2−/−* males.* Insulin-like growth factor 2* (*Igf2*) was downregulated in female* Id2−/−* mice, while* Fbp1*, a rate-limiting enzyme in gluconeogenesis, and* Shc1*, a component in the IGF-1-regulated pathway, were upregulated. For adipogenesis,* bone morphogenetic protein 4* (*Bmp4*) was elevated 1.7-fold (n.s.) in male and 1.6-fold in female* Id2−/−* mice. Consistent with the insulin signaling array,* Irs1* was elevated in* Id2−/−* mice.* Nuclear receptor coactivator 2* (*Ncoa2*),* PR domain containing 16* (*Prdm16*),* Pparα*, and* twist homolog 1 *(*twist1*) were downregulated, in grouped analysis of male and female* Id2−/−* mice.* Fatty acid synthase* (*Fasn*),* lipase*,* hormone sensitive* (*Lipe*), and* Peroxisome proliferative activated receptor*,* gamma*,* coactivator 1 beta* (*Ppargc1β*/*PGC-1β*) were all downregulated in male* Id2−/−* mice. Female* Id2−/−* mice displayed a downregulation of* proliferative activated receptor*,* gamma*,* coactivator 1 alpha* (*Ppargc1α/PGC-1α*). A small 1.2-fold downregulation of* Peroxisome proliferator activated receptor gamma* (*Pparγ*) was detected in* Id2−/−* males, where the *p* value was approaching significance (*p* = 0.061). Note that the thermogenic protein,* uncoupling protein 1* (*ucp1*), was present on both the insulin signaling and adipogenesis arrays, but its levels of expression were not significantly altered in the* Id2*−/− mice.

## 4. Discussion

In the present study, we discovered a reduced core body temperature in* Id2*−/− mice, and this effect was not found to be dependent upon the time-of-day. Moreover, from the iBAT of* Id2−/−* mice, genes involved in insulin signaling and adipogenesis were differentially regulated in a sex-dependent manner. These results reveal a role of* Id2* in the regulation of thermogenesis and BAT metabolic functions.

Our previous study revealed that* Id2−/−* mice exhibit less activity as demonstrated by daily counts of general activity and the wheel running activity, which could partially explain the reduced core body temperature, since less physical activity would generate less heat [10]. Moreover,* Id2−/−* mice show a reduced body mass and less gonadal adipose deposits [6, 10, 11]. As the subcutaneous and intradermal fat functions as thermal insulation for mice to preserve heat loss,* Id2−/−* mice with low fat content might tend to lose heat more readily than WT mice. Furthermore, the reduced body temperature associated with lower fat content might contribute to the high death rate observed previously (mice housed under normal temperature), which was rescued by a high fat diet that resulted in increased total body fat [11]. Specifically in male* Id2−/−* mice iBAT we observed increased glucose uptake and reduced TG accumulation [10], suggesting alterations in its metabolic programing. Additionally, both male and female* Id2−/−* mice exhibited an increased activated iBAT volume [10]. Interestingly, our results suggest that the role of* Id2* in thermoregulation is opposite to the function of another member of this HLH family,* Id1*, whose deficiency results in higher thermogenesis and an elevated BAT expression of thermogenic proteins [17]. Notably,* Id1* has a distinct and opposite function in WAT adipogenesis compared to* Id2*, despite both* Id1* and* Id2* null mice exhibiting reduced adiposity [10–12, 17]. Lastly, we examined the relationship between body mass and body temperature in* Id2−/−* mice by regression analysis and revealed a limited relationship between the two variables [18]. No significant relationship was observed between body mass and body temperature at any time of the day or in the two sexes. However, as can be seen with the *y*-intercept of the regression lines, both* Id2*−/− male and female mice expressed a consistently lower temperature compared to WT controls, irrespective of body mass, and this feature was observed during both the day and night phases of the LD cycle. These results suggest a role for* Id2* in the regulation of core body temperature.

In this study we also measured iBAT mass and iBAT/body mass ratio. While there was a tendency for higher iBAT/body mass in both* Id2−/−* male and female mice, this was not determined to be a significant difference. Note that the average body mass of* Id2*−/− mice used for both the iBAT weight and body temperature experiments was found to be significantly lower, consistent with our previous studies [7, 10]. Important is the fact that a lower body mass, found for some of the* Id2*−/− mice and for males in particular, does not correlate with a lower body temperature, and body mass in this situation is therefore an independent factor when predicting core body temperature.

It is important to note that while the objective of examining body temperature using the implanted thermometers was to record “core” body temperature, the position of the implants may not give an* exact* measure of true core body temperature. However, in a comparable study of mouse body temperatures, temperature measurements were similar whether derived from similarly subcutaneously implanted thermometers in the interscapular region of WT and* Rev-erbα* mutant mice or as determined using dataloggers that were implanted within the abdomen [3].

*Id2* is rhythmically expressed in BAT [4, 15] amongst other tissues [6, 7]. ID2 protein has also been observed to be rhythmic in its abundance over the 24 hr diurnal/circadian cycle within the liver and heart [6] (Ward, Fernando, Hou, and Duffield, unpublished data). A role for ID2 has been established as a mediator of circadian clock output and control of expression patterns of clock controlled genes (CCGs) within the liver [6]. CCGs encompass ~10% transcriptome in individual tissues [19]. It is for this reason that we examined whether any of the genes identified as differentially expressed in iBAT were in fact known CCGs. Using the CIRCA database [14, 15], 5 of the 17 differentially genes associated with adipogenesis were found to be CCGs (e.g.,* Pparα* and* PGC-1α*), and so a possible role for ID2 is in mediating circadian regulatory effects on these genes within BAT. However, further investigation would be required to test this hypothesis. The observation that few of the differentially regulated genes involved in insulin signaling are CCGs (1 out of 13 genes) suggests that the contribution of ID2 to insulin signaling intrinsic to BAT is independent of the role of ID2 in mediating circadian clock output [6].

**Figure 3:**
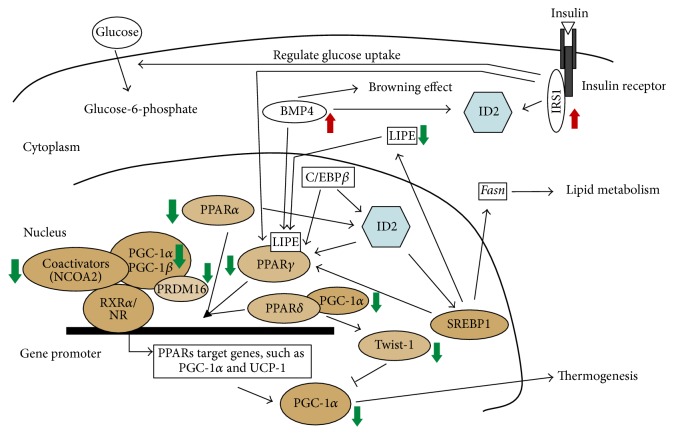
*Id2* network model in insulin signaling, thermogenesis, and adipogenesis pathways. A schematized and partial view of the signaling pathway indicating its major downstream targets and factors susceptible to interfering with signaling. Function of ID2 is not necessarily limited to the nucleus [8]. ↑ or ↓, up- or downregulation of gene expression, respectively, as determined by PCR array analysis in the current study.

In order to explain how* Id2* deficiency has an impact on BAT insulin signaling and adipogenesis, we propose a network model (Figure 3). The nuclear receptor PPARs are fundamentally important for energy homeostasis and* Id2* plays a role in interfacing with the molecular pathways upstream or downstream of these transcriptional factors. Expression of two members of the PPAR subfamily of ligand-activated nuclear receptors,* PPARα* and* PPARγ*, was downregulated in our study. PPAR*α* is highly expressed in BAT and considered a marker of BAT; it also plays an important role in the overall regulation of lipid metabolism; and its target genes are involved in mitochondrial and peroxisomal *β*-oxidation of fatty acids (FAs) [20–22]. Moreover, PPAR*α* regulates the expression of uncoupling protein 1 (*ucp1*), which confers on BAT its thermogenic capacity [23].* PGC-1α* (downregulated in our study) is a transcriptional coactivator involved in the control of energy metabolism and critical for BAT thermogenesis and enhancing overall mitochondrial oxidative activity [24]. PPAR*α* can induce* PGC-1α* gene expression and contributes to the thermogenic activation of brown fat [25]. PRDM16 exhibits a brown fat selective expression pattern and regulates the thermogenic gene program in brown and beige adipocytes [26]. The observation of reduced* Prdm16* expression in* Id2*−/− mice is consistent with the role of PRDM16 as a transcriptional regulator of PGC-1*α* [27]. Likewise, studies have demonstrated the linkage between* Id2* and PPAR*α* [28]. PPAR*γ* is essential for adipocyte differentiation, and PPAR*γ* alone generates a fat phenotype that is common to both WAT and BAT. The CCAAT enhancer binding protein beta (C/EBP*β*) and PGC-1*α* are critical for controlling PPAR*γ* expression in BAT and for determining BAT-specific programs [29, 30]. The PPAR*γ* thermogenic effect in BAT is mediated by PGC-1*α* [24]. It has been observed that overexpression of* Id2* associates with* PPARγ* expression, ID2 acts upstream of PPAR*γ*, and C/EBP*β* induces* Id2* expression during the adipogenesis process [12, 31]. Cofactors such as NCoA2 (downregulated in our study) can interact directly with PPAR*γ* to initiate its own transactivation [32]. Moreover, LIPE (downregulated in our study) could modulate adipose metabolism by reducing the availability of ligands for PPAR*γ*, since gene knockout of LIPE in mice attenuates activation of PPAR*γ* [33]. LIPE is also able to hydrolyze stored TGs in adipose tissue and to mobilize free FA from adipose tissue [34]. Furthermore, PPAR*γ* is a direct target of the transcription factor sterol response element binding protein 1 (SREBP1), whose transcriptional activity is modulated by ID2 and which regulates downstream lipid metabolism genes such as* lipe* and* Fasn* [35, 36]. Additionally,* Irs1* (upregulated in our study) plays essential roles in the differentiation of brown adipocytes and expression of PPAR*γ* [37, 38]. Previous studies have revealed IRS1-regulated* Id2* gene expression [39], although in the current study it is unclear whether this is a direct effect or a feedback response. As for the mechanism by which IRS1 is elevated in* Id2*−/− iBAT, it is unclear and warrants further investigation. BMP4 (upregulated in our study) is able to induce the white to brown transition of adipose cells, which could indirectly regulate PPAR*γ* activation [40, 41]. The elevated BMP4 expression in the context of reduced PPARs is surprising since BMP4 upregulation is associated with increased BAT adipogenesis and the WAT browning effect [42]. Interestingly, the Id2 gene promoter has BMP-response elements and has been shown to be a target of BMP signaling [43]. PPAR*δ* plays an integral role in transcriptional network regulation of fat-burning genes and brown fat metabolism. PGC-1*α*/PPAR*δ* could regulate brown fat metabolism through Twist-1 tuning [44].* Twist-1* (downregulation in our study) encodes a basic HLH transcription factor, and overexpression of* Twist-1* is associated with* Id2* expression [45].

Note that the gene encoding the thermogenic protein, UCP1, was present on the PCR arrays, but no difference in its expression was observed between genotypes of either sex. Interestingly, in the* Id1* null mouse,* ucp1* gene expression is elevated in iBAT, and this is associated with an* increased* core body temperature phenotype [17]. Thus, it is surprising that in the* Id2*−/− mouse that exhibits a* reduced* body temperature phenotype we do not observe a reduction in* ucp1* gene expression. Of course, this does not exclude, however unlikely, the possibility of an altered UCP1 protein abundance through a posttranscriptional/posttranslational process.

Hypoxia, while not a focus of the current study, is known to reduce body temperature in mammals and contribute to the thermogenic activity of BAT [46]. It is noteworthy that in a recent study of human glioblastoma cells/tissue, an important role was established for ID2 in modulating the cellular effects of hypoxia and its activation of the HIF2*α* pathway [47]. The Id2 gene is also a target for HIF1*α* and HIF2*α* [47, 48], making it part of a positive feedback loop mechanism, at least in models of brain tumor. It is plausible that the hypoxic effects on BAT function might also include a contribution from ID2, and this would be an important pathway to examine in future experiments in this context.

It is a somewhat contradictory finding that elements of the thermogenic pathway are reduced (e.g.,* PGC1-α*) or unaltered (*ucp1*) in* Id2*−/− mice but that* Id2*−/− male iBAT exhibits increased glucose uptake (PET-FDG) coupled with reduced iBAT triglyceride levels and a systemwide enhanced insulin sensitivity [10, 11] and that core body temperature is consistently reduced in both* Id2*−/− male and female mice.* Id2*−/− mice also exhibit an altered 24 hr locomotor activity and feeding profile and an overall reduction in nocturnal locomotor activity [10], the latter of which suggests a reduction in energy expenditure/increased energy conservation. Clearly the BAT adipogenic program is altered in both male and female* Id2*−/− mice, as is WAT adipogenesis [10–12], and it is likely that a change in BAT function contributes to the reduced temperature phenotype. Due to the nature of whole body knockout of ID2, it is possible that the temperature phenotype and iBAT gene changes observed are secondary to whole body metabolic changes. Clearly additional experiments are required to elucidate the relative contributions of these and other potential components in generating the altered core body temperature phenotype.

Our previous study showed enhanced glucose uptake in iBAT of* Id2*−/− mice [10], and the present study reveals a reduced core body temperature in* Id2*−/− mice. This discrepancy could partially be explained by the differential regulation of* Irs1*,* Lipe*,* PPAR*s, and* PGC-1*s. It has been reported that degradation of IRS1 leads to impaired glucose uptake in adipose tissue [49]. Therefore, upregulation of IRS1 might explain the increased glucose uptake we observed before [10], whereas downregulation of LIPE, PPARs, and PGC-1s might contribute to reduced FA oxidation, impaired adipogenesis, and a lower body temperature. Inactivation of PPARs is associated with insulin resistance [50, 51], yet paradoxically* Id2−/−* mice show enhanced insulin sensitivity with downregulated PPARs [10, 11]. It has been suggested that mice that lack one allele of the PPAR*γ* gene are more sensitive to insulin, which could partially explain the enhanced insulin sensitivity we observe in* Id2* null mice [10, 51]. Furthermore, the differential regulation of genes specifically in female mutant mice, such as* Fbp1*, a rate-limiting enzyme in gluconeogenesis,* Shc1*, a component in the IGF-1-regulated pathway, and* Igf2*, suggests a sex-specific physiological program for ID2 in BAT.

## 5. Conclusion

*Inhibitor of DNA binding 2* is rhythmically expressed in BAT [4, 15], and the observation that few of the differentially regulated genes involved in insulin signaling are CCGs suggests that the contribution of ID2 to insulin signaling intrinsic to BAT is independent of the role of ID2 in mediating circadian clock output [6]. Overall, ID2 seems to be an important coordinator of energy homeostasis including insulin signaling, adipogenic programing, and thermoregulation. In conclusion, our finding that ID2 contributes to the regulation of body temperature and energy homeostasis presents the possibility that ID2 could be a potential therapeutic target for metabolic disease. Further, these data emphasize the influence of* Id2* on BAT molecular signaling and physiology in a sex-specific manner.

## Supplementary Material

Supplementary Table 1 summarizes the regression analysis of time-of-day representative core body temperatures (day and night).
Supplementary Table 2 shows the mean ± SEM body mass of wild type and Id2−/− mice.

## Figures and Tables

**Table 1:** Differentially expressed genes from insulin signaling pathway of *Id2−/−* mice. Genes with significant differences (*p* < 0.05) are shown in bold. Peak phase value (determined by Lomb-Scargle phase values within CIRCA) in circadian time (CT) is provided where gene was identified as a clock control gene. Rhythmic genes identified from CIRCA database of Mouse 1.OST Brown Adipose (Affymetrix).

Insulin signaling array	Symbol	Refseq	All		Male		Female		Clock control gene-peak phase
			Fold change	*p* value	Fold change	*p* value	Fold change	*p* value	
*Gene name*									
AE binding protein 1	**Aebp1**	NM_009636	1.9	0.09			**1.6**	**0.03**	—
Complement factor D (adipsin)	**Cfd**	NM_013459	**2.4**	**0.01**	**3.6**	**0.03**			—
Fructose bisphosphatase 1	**Fbp1**	NM_019395	1.3	0.22			**1.6**	**0.05**	—
Glucose-6-phosphatase, catalytic	**G6pc**	NM_008061	1.5	0.28	1.5	0.33	**1.6**	**0.02**	—
Growth factor receptor bound protein 10	**Grb10**	NM_010345	1.5	0.08					—
Insulin receptor substrate 1	**Irs1**	NM_010570	**1.4**	**0.01**	1.3	0.07	**1.4**	**0.05**	—
Protein tyrosine phosphatase, receptor type, F	**Ptprf**	NM_011213	**1.7**	**0.05**			**2.6**	**0.05**	—
Scr homology 2 domain containing transforming protein C1	**Shc1**	NM_011368					**1.25**	**0.01**	—

Glucose-6-phosphatase, catalytic, 2	**G6pc2**	NM_021331			−3.2	0.08			—
Insulin-like growth factor 2	**Igf2**	NM_010514	−1.2	0.21			**−1.4**	**0.04**	—
V-Ki-ras2 Kirsten rat sarcoma viral oncogene homolog	**Kras**	NM_021284	−1.1	0.09	**−1.25**	**0.03**			—
Protein kinase C, iota	**Prkci**	NM_008857	**−1.2**	**0.02**	**−1.3**	**0.02**			CT0
Thyroglobulin	**Tg**	NM_009375	−1.8	0.29	**−3.5**	**0.02**			—

**Table 2:** Differentially expressed genes from adipogenesis pathway of *Id2−/−* mice. Genes with significant differences (*p* < 0.05) are shown in bold.

Adipogenesis array	Symbol	Refseq	All		Male		Female		Clock control gene-peak phase
			Fold change	*p* value	Fold change	*p* value	Fold change	*p* value	
*Gene name*									
Bone morphogenetic protein 4	**Bmp4**	NM_007554	**1.6**	**0.04**	1.7	0.12	**1.6**	**0.01**	—
Complement factor D (adipsin)	**Cfd**	NM_013459	**2.5**	**0.02**	**4.3**	**0.03**			—
Insulin receptor substrate 1	**Irs1**	NM_010570	**1.2**	**0.03**	1.1	0.29	**1.4**	**0.02**	—
TSC22 domain family, member 3	**Tsc22d3**	NM_010286	**1.6**	**0.04**	1.9	0.09			CT16

Axin 1	**Axin1**	NM_009733	−1.2	0.07	**−1.3**	**0.05**			—
Cyclin-dependent kinase 4	**Cdk4**	NM_009870	**−1.4**	**0.003**	**−1.7**	**0.001**			CT6
Delta-like 1 homolog (Drosophila)	**Dlk1**	NM_010052	**−1.9**	**0.01**	**−2.2**	**0.01**			—
Fatty acid synthase	**Fasn**	NM_007988	−1.2	0.06	**−1.4**	**0.02**			—
Lipase, hormone sensitive	**Lipe**	NM_010719	−1.2	0.06	**−1.5**	**0.02**			—
Nuclear receptor coactivator 2	**Ncoa2**	NM_008678	−1.2	0.07	−1.3	0.07			—
Peroxisome proliferator activated receptor alpha	**Ppara**	NM_011144	**−2.1**	**0.01**	−2.3	0.06	−1.7	0.09	CT6
Peroxisome proliferator activated receptor gamma	**Pparg**	NM_011146			−1.2	0.06			
Peroxisome proliferative activated receptor, gamma, coactivator 1 alpha	**Ppargc1a** **(PGC-1** **α** **)**	NM_008904	−1.2	0.09			**−1.5**	**0.004**	CT8
Peroxisome proliferative activated receptor, gamma, coactivator 1 beta	**Ppargc1b** ** (PGC-1** **β** **)**	NM_133249	**−1.4**	**0.01**	**−1.4**	**0.04**			CT5
PR domain containing 16	**Prdm16**	NM_027504	**−1.3**	**0.03**	**−1.5**	**0.03**			—
Twist homolog 1 (Drosophila)	**Twist1**	NM_011658	**−1.8**	**0.01**	−2.0	0.06	**−1.5**	**0.04**	—
Actin, beta	**Actb**	NM_007393	**−1.2**	**0.02**	**−1.4**	**0.004**			—

## Additional Points

Supplementary Table  1 summarizes the regression analysis of time-of-day representative core body temperatures (day and night). Supplementary Table  2 shows the mean ± SEM body mass of wild type and* Id2−/−* mice.
