# Supplementary material for: Impaired Thermogenesis and a Molecular Signature for Brown Adipose Tissue in Id2 Null Mice
Source: J Diabetes Res. 2016 Apr 10;2016:6785948. doi: 10.1155/2016/6785948 (PMC4842059; doi:10.1155/2016/6785948)
Supplement: Supplementary file 1 — Supplementary Table 1 summarizes the regression analysis of time-of-day representative core body temperatures (day and night). Supplementary Table 2 shows the mean ± SEM body mass of wild type and Id2−/− mice. [file 6785948.f1.zip › mat.6785948.v2.pdf]

Table S1

Regression analysis of time-of-day representative core body temperatures (day and night).

	Normal ambient temperature (21°C)							
	Male				Female			
	ZT5.5		ZT20.5		ZT5.5		ZT17.5	
	WT	*Id2-/-*	WT	*Id2-/-*	WT	*Id2-/-*	WT	*Id2-/-*
y-intercept (°C)	37.7	36.4	37.1	37.1	37.6	35.8	37.5	35.9
R^2	0.06	0.001	0.04	0.0001	0.006	0.08	0.006	0.21
Slope	-0.016	-0.007	0.021	0.002	-0.008	0.056	0.009	0.093
	**Thermoneutral temperature (30°C)**							
	Male				Female			
	ZT5.5		ZT17.5		ZT5.5		ZT17.5	
	WT	*Id2-/-*	WT	*Id2-/-*	WT	*Id2-/-*	WT	*Id2-/-*
y-intercept (°C)	38.2	37.9	38.3	36.9	38.1	36.9	38.4	38.1
R^2	0.07	0.07	0.02	0.02	0.07	0.0001	0.03	0.03
Slope	-0.027	-0.045	-0.009	-0.026	-0.039	0.003	-0.018	-0.034

Table S2

Mice Body Weights (g) (Mean ± SEM)							
iBAT Weight Experiment				Temperature Experiment			
Male		Female		Male		Female	
WT (n=15)	*Id2-/-* (n=7)	WT (n=10)	*Id2-/-* (n=9)	WT (n=19)	*Id2-/-* (n=20)	WT (n=17)	*Id2-/-* (n=17)
32.76 ± 1.03	23.84 ± 0.64 ***	25.84 ± 1.02	19.83 ± 1.66 **	31.72 ± 1.67	24.58 ± 1.25 ***	21.65 ± 1.29	19.42 ± 0.94
Two-Factor ANOVA							
Genotype: *p*<0.001; Sex: *p*<0.001; Interaction: n.s.<br>Tukey post hoc tests for intra-sex differences, ***p*<0.01, ****p*<0.001				Genotype: *p*<0.001; Sex: *p*<0.001; Interaction: n.s.<br>Tukey post hoc tests for intra-sex differences, ****p*<0.001			
