# Supplementary material for: Impaired Thermogenesis and a Molecular Signature for Brown Adipose Tissue in Id2 Null Mice
Source: J Diabetes Res. 2016 Apr 10;2016:6785948. doi: 10.1155/2016/6785948 (PMC4842059; doi:10.1155/2016/6785948)
Supplement: Supplementary file 1 — Supplementary Table 1 summarizes the regression analysis of time-of-day representative core body temperatures (day and night). Supplementary Table 2 shows the mean ± SEM body mass of wild type and Id2−/− mice. [file 6785948.f1.zip › Supplementary description.docx]

**Supplementary Material**

Supplementary Table 1 summarizes the regression analysis of time-of-day representative core body temperatures (day and night). Supplementary Table 2 shows the mean ± SEM body mass of wild type and *Id2*-/- mice.
